# Capsaicin has an anti-obesity effect through alterations in gut microbiota populations and short-chain fatty acid concentrations

**DOI:** 10.29219/fnr.v64.3525

**Published:** 2020-02-19

**Authors:** Yuanwei Wang, Cheng Tang, Yong Tang, Haiyan Yin, Xiong Liu

**Affiliations:** 1 College of Food Science, Southwest University, Chongqing, China; 2 College of Life Science and Technology, Southwest Minzu University, Chengdu, China; 3 College of Medicine, Chengdu University, Chengdu, China; 4 School of Acupuncture, Chengdu University of Traditional Chinese Medicine, Chengdu, China

**Keywords:** anti-obesity, capsaicin, gut, microbiota, short-chain fatty acids, food intake

## Abstract

**Background:**

Capsaicin (CAP) has an anti-obesity effect that has been shown to involve the transient receptor potential vanilloid-1 (TRPV1) channel. Importantly, recent studies in high fat diet (HFD)-fed mice show that CAP also alters gut microbiota composition and causes weight loss in HFD-fed mice. Many studies have suggested that short-chain fatty acids (SCFAs) mediate the links between diet, gut microbiota, and fat storage.

**Objective:**

The present study investigated whether CAP exerted its anti-obesity effect through changes in the composition of gut microbiota and SCFAs, and whether the TRPV1 contributes to CAP’s effects against obesity in HFD-fed mice.

**Design:**

C57BL/6J (TRPV1+/+) and B6.129X1-Trpv1tm1Jul/J (TRPV1-/-) mice were respectively divided into three groups (*n* = 6),that is SLD, HFD-fed, and CAP (2 mg/kg, po) +HFD fed and were administered respective treatment for 12 weeks.

**Results:**

We observed significantly lower weight gain and food intake, triglyceride, cholesterol, glucose, and insulin levels in HFD+CAP-fed TRPV1knockout (KO) mice compared to the HFD-fed KO mice, though this effect was more obvious in wild-type (WT) mice. CAP increased the numbers of *Akkermansia*, *Prevotella*, *Bacteroides*, *Odoribacter, Allobaculum, Coprococcus,* and S24-7, and reduced the numbers of *Desulfovibrio*, *Escherichia, Helicobacter*, and *Sutterella* in the HFD+CAP-fed WT and KO mice compared with HFD-fed WT and KO mice. CAP increased the relative abundances of SCFAs producing the bacterial species, which increased intestinal acetate and propionate concentrations, which were beneficial in prevention and treatment of obesity.

**Conclusions:**

Results from our study indicate that the reduced food intake and anti-obesity effect of CAP had been observed regardless of TRPV1 channel activation, and which is mediated by changes in the gut microbiota populations and SCFAs concentrations.

## Popular scientific summary

- Anti-obesity effect of CAP has been observed regardless of TRPV1 channel activation.
- CAP stimulates the secretion of SCFA by regulating gut microbiota.
- CAP has an anti-obesity effect by causing the reduction in food intake.
- CAP reduces the relative abundance of LPS-producing *Proteobacteria* spp.

Obesity is one of the most important health problems worldwide (1). The incidence of obesity has been increasing for many years; as a result of improvements in living standards, lifestyle changes, excessive calorie intake, and physical inactivity, the incidence of obesity has been increasing for many years. It is reported that global obesity rose from affecting 105 million people in 1975 to 641 million in 2014 (1). Many anti-obesity medicines have been developed, but their use is associated with side effects (2); therefore, dietary measures remain the fundamental strategy for the prevention and treatment of obesity and its related metabolic disorders (3). Some natural food materials have been studied as potential agents for the prevention or treatment of obesity (4). Ingestion of capsaicin (CAP), the active ingredient in chili peppers, has long been associated with a lower risk of obesity, and the mechanisms of this effect have been extensively studied in rodents and other species. Among the potential molecular mechanisms, the activation of transient receptor potential vanilloid-1 (TRPV1) cation channels appears to be critical (5, 6).

The gut microbiota, composed of trillions of microorganisms, is now accepted as an integral part of the metabolome, in a ‘super-organismal’ context (7). Many studies have shown that altered microbial composition is associated with the development of obesity and metabolic syndrome in humans (8). These findings suggest that targeting of the gut microbiota may represent a new strategy for both the prevention and treatment of obesity and metabolic syndrome.

Short-chain fatty acids (SCFAs) such as acetate, butyrate, and propionate are the major products of dietary fiber fermentation by the gut microbiome. Recent studies have suggested that the molecular mechanism underpinning the relationships between diet, gut microbiota, and fat storage involves SCFAs (9). Many clinical studies have also shown that SCFAs can be used for the treatment of obesity (9–11). Therefore, it has been hypothesized that SCFAs play a key role in the prevention and treatment of the metabolic syndrome.

Recently, Baboota et al. demonstrated using quantitative polymerase chain reaction methods (qPCR), that oral administration of CAP alters gut microbiota composition and causes weight loss in high-fat diet (HFD)-fed mice (12). Shen et al. and Kang et al. further explored the effects of CAP on gut microbiota in HFD-fed mice using high-throughput sequencing (13, 14). However, it is unknown whether the anti-obesity effect of CAP was due to an altered gut microbiota composition regardless of TRPV1 channel activation. Alterations to the composition of gut microbiota could affect SCFA production, which in turn could affect whole body metabolism. It is not clear what changes in SCFAs were caused by CAP and how CAP affects them. In our study, we measured gut microbiota composition by using high-throughput sequencing and SCFA composition by using gas chromatography-mass spectrometry (GC-MS) in wild-type (WT) and TRPV1 knockout (KO) mice fed a standard lipid diet (SLD), a HFD, or an HFD plus intragastrical administration of CAP (HFD+CAP), to explore the effects of CAP on the gut microbiota, SCFA production, and host energy metabolism with and without TRPV1 cation channel activation.

## Materials and methods

### Materials

CAP (M2028, ≥95%) was purchased from Sigma-Aldrich, Inc. (St. Louis, MO, USA). Standard lipid diet (SLD, D12450B, 10% calories as fat) and high-fat diet (HFD, D12492, 60% calories as fat) were purchased from Research Diets (New Brunswick, NJ, USA). All other reagents were of the highest commercially available grade.

### Animals and experimental design

Animal experiments were carried out in strict accordance with the recommendations provided in the Guide for the Care and Use of Laboratory Animals and were approved by the Ethics Committee of Laboratory Animal Management Committee of Sichuan Province, China. (Approval number: SYXK-2014-189). Eight-week-old female C57BL/6J WT and B6.129X1-Trpv1^tm1Jul^/J (TRPV1^−/−^; KO) mice were housed under standard conditions (temperature, 22 ± 2°C; humidity, 55 ± 5%), with free access to food and water and a 12 h light/dark cycle.

After a 1-week acclimation and the feeding of an SLD for 2 weeks, WT and KO mice were each randomly divided into three groups (*n* = 6): an SLD group, an HFD group, and an HFD+CAP group, keeping one mice per cage in the animal facility. Each mouse in the HFD+CAP group was given 2 mg/kg body mass CAP intragastrically by dissolving in 0.9% saline containing 3% ethanol and 10% Tween-80 on alternate days, and SLD and HFD-fed mice were given the corresponding vehicle, for 12 weeks.

Body mass and food intake were recorded weekly. Feces were collected weekly and stored at −80°C. At the end of the feeding period, plasma, feces, hypothalamus, and colon were collected after a 12-h fast, and the animals were sacrificed by cervical dislocation after CO2 exposure.

### Biochemical analysis

Plasma triglyceride (TG) and cholesterol concentrations were determined using commercially available kits (BioSino, Beijing, China). Plasma insulin concentration was measured using a mouse insulin ELISA kit (Mercodia, Uppsala, Sweden).

### Glucose tolerance tests

After 11 weeks of treatment, mice were fasted overnight (9 h). They were then gavaged with 1 g/kg body mass D-glucose solution and their blood glucose concentrations were measured using a glucometer (ACCU-CHEK Aviva meter, Roche, USA), 0, 15, 30, 60, and 120 min afterwards.

### Gut microbiota analysis by 16S rRNA gene sequencing

Total genomic DNA was extracted from feces using a QIAamp DNA Stool Minikit (Qiagen, Hilden, Germany), following the manufacturer’s instructions. The genomic DNA concentration was normalized to 1 ng/μL. The V4 region of the 16S rRNA gene was amplified using a 515F forward primer (5’-GTGCCAGCMGCCGCG GTAA-3’) and an 802R reverse primer (5’-GGACTACHVGGGTWTCTAAT-3’). PCR reactions were performed using 30 ng purified DNA, 12.5μL Phusion High-Fidelity PCR master mix (NEB, USA), 100 nmol/L forward and reverse primers, and nuclease-free water to a final volume of 25 μL. PCR cycling conditions consisted of an initial denaturation of 1 min at 98°C, 30 cycles of 30 s at 98°C, 30 s at 58°C, and 30 s at 72°C, and then a final 10 min at 72°C. The PCR products were purified using AmpureXP beads (Agencourt) and then sent to the Beijing Genomics Institute (Shenzhen, China) for sequencing using an Illumina MiSeq PE250 sequencer.

Data analysis was performed by using Quantitative Insights Into Microbial Ecology (QIIME, v1.80). Clean data was obtained from sequence reads by pre-processing the removal of the primer sequence, truncating sequence reads that did not have an average quality of 20 over a 30 bp sliding window according to the Phred algorithm, and by removing trimmed reads of <75% of their original length, as well as by using paired reads. These stringent criteria allowed retention of nearly 94% of the reads. FLASH, a fast-computational tool that extends the length of short reads by overlapping paired-end reads in genome assemblies, was also used. For quality control purposes, no mismatches were allowed in the primer or barcode regions. Furthermore, tags with ambiguous bases (N) and screened potential chimeras were removed. To focus the analysis on bacterial taxa, non-chimeric sequences were mapped into operational taxonomic units (OTUs) using USEARCH, and 97% of OTUs were picked using a closed-reference OTU picking protocol against the Ribosomal Data Project II database. Reads that did not match a reference sequence with >97% identity were discarded. Richness and diversity indices (observed species, Chao, abundance-based coverage estimator (ACE), and Shannon) and dissimilarity matrices (Bray-Curtis and weighted UniFrac) were estimated using Mothur software.

### Fecal short-chain fatty acid quantification by GC-MS

Quantification analysis of fecal SCFAs was undertaken as previously described using an Agilent 7890 A gas chromatograph coupled to an Agilent 5975C mass spectrometer (Agilent Technologies, USA). Fecal samples were homogenized in 0.005 M aqueous NaOH and then centrifuged at 13,200 *g* at 4°C for 20 min. The supernatant was derivatized using a proh/pyridine mixture (3:2, v/v) and propyl chloroformate, and the derivatives were extracted in two stages using hexane. The concentrations of the SCFAs (acetic acid, propionic acid, butyric acid) were measured using a polar DB-WAX capillary column (30 m × 0.25 mm, 0.25 μm film thickness; Agilent, CA). Helium was used as the carrier gas at a constant flow rate of 1 mL/min. The initial oven temperature was maintained at 60°C for 5 min, ramped to 250°C at 10°C/min, and finally kept at this temperature for 5 min. The temperatures of the front inlet, transfer line, and electron impact ion source were set at 280°C, 250°C, and 230°C, respectively. Data handling was performed by using Agilent MSD ChemStation (E.02.00.493, Agilent Technologies, Inc., USA).

### RNA isolation and quantification of gene expression

Total RNA was isolated from mouse hypothalamus and colon using Ribopure RNA extraction kits (Invitrogen, USA) according to the manufacturer’s instructions. cDNA synthesis was performed using a Super Script III First-Strand Synthesis System for RT-PCR Kit (Invitrogen, Rockville, MD) and 1μg total RNA. cDNA synthesized from total RNA was evaluated using a real-time quantitative PCR system (CFX96 Touch™ Real-Time PCR Detection System, Bio-Rad, USA). The primers for the genes targeted in this study are listed in Table 1. Data were analyzed using the ΔΔCt method (SABiosciences, Qiagen, USA), with normalization of gene expression using the geometric mean of four reference genes (β-actin or 18s RNA).

**Table 1:** The primers used for real-time RT-PCR.

Gene	Primer	Sequence	References
POMC	sense	ATAGATGTGTGGAGCTGGTG	(15)
	antisense	GGCTGTTCATCTCCGTTG	
CART	sense	GCGCTATGTTGCAGATCGAA	(15)
	antisense	TCACACAGCTTCCCGATCCT	
AGRP	sense	CAGACCGAGCAGAAGAAG	(15)
	antisense	GACTCGTGCAGCCTTACA	
NPY	sense	CCGCTCTGCGACACTACAT	(16)
	antisense	TGTCTCAGGGCTGGATCTCT	
FFAR2	sense	ACAGTGGAGGGGACCAAGAT	(11)
	antisense	GGGGACTCTCTACTCGGTGA	
GLP-1	sense	CAAACCAAGATCACTGACAAGAAAT	(11)
	antisense	GGGTTACACAATGCTAGAGGGA	
PYY	sense	CTTCACAGACGACAGCGACA	(11)
	antisense	GGGAAATGAACACACACAGCC	
PYY-Y2	sense	TCCGGGAATACTCCCTGATTG	(17)
	antisense	GCAAAACGTACAGGATGAGCAG	
GLP-1R	sense	AGTAGTGTGCTCCAAGGGCAT	(18)
	antisense	AAGAAAGTGCGTACCCACCG	
β-actin	sense	TTGTAACCAACTGGGACGATATGG	(15)
	antisense	GATCTTGATCTTCATGGTGCTAGG	
18s RNA	sense	AGGATGTGAAGGATGGGAAG	(11)
	antisense	TTCTTCAGCCTCTCCAGGTC	

### Statistical analysis

The comparison of difference among diet groups was performed using one-way analysis of variance (ANOVA) followed by Dunnett’s test. All data are presented as the mean ± SD. *P* values <0.05 were considered to be statistically significant. Principal coordinate analyses (PCoAs) were used when analyzing differences in bacterial community structures. Nonparametric ANOVA with false discovery rate (FDR) correction was used in analyzing the relative abundances of the bacterial taxa. GraphPad Prism version 7.0 (GraphPad Software, San Diego, CA) was used in all analyses.

## Results

### CAP has an anti-obesity effect regardless of TRPV1 channel activation

**Fig. 1:**
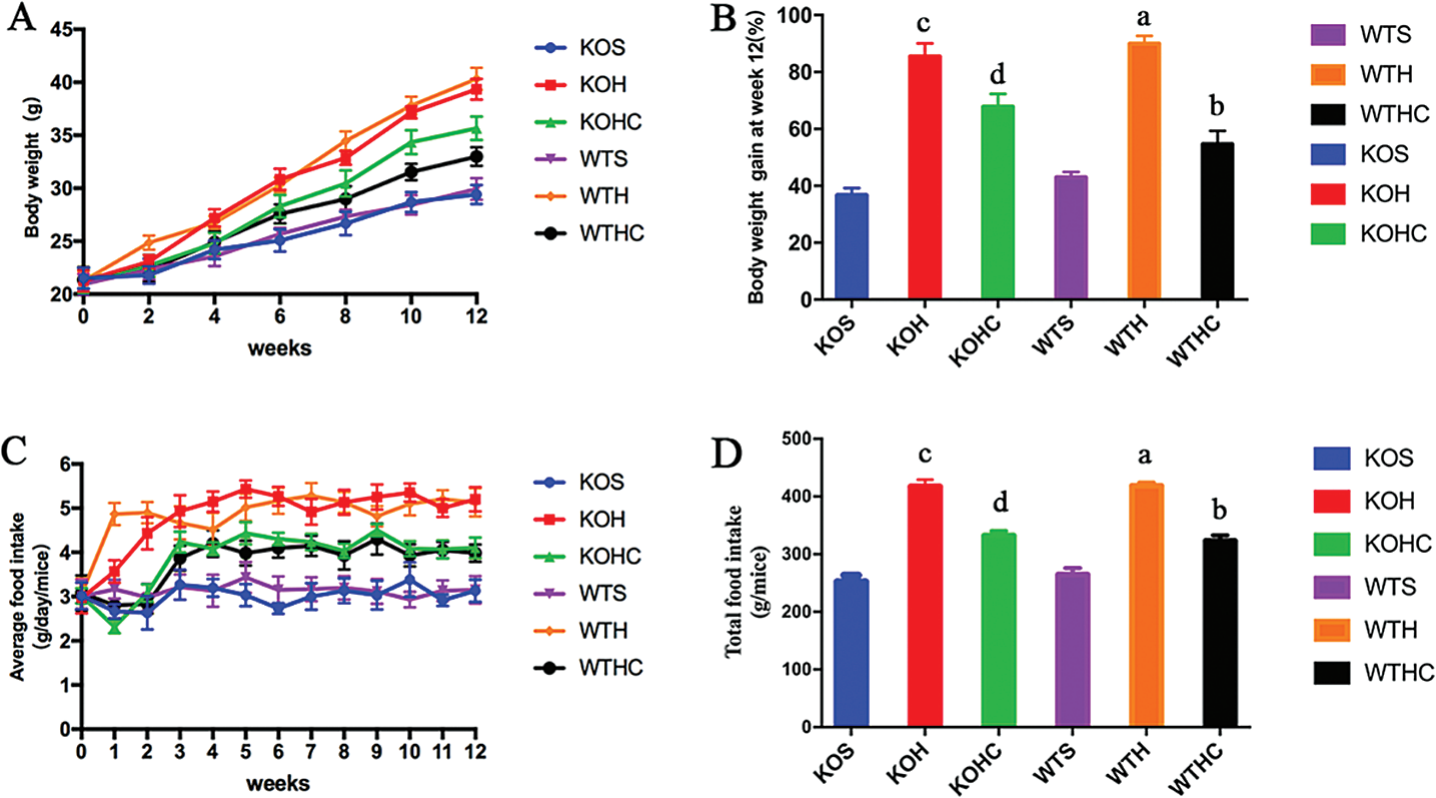
CAP protect against the high-fat diet induced increases in body weight. WTH, WT mice fed with high-fat diet; WTHC, WT mice fed with high-fat diet and CAP; KOS, KO mice fed with low fat diet; KOH, KO mice fed with high-fat diet; KOHC, KO mice fed with high-fat diet and CAP. (A) Gained body weight within12 weeks; (B) relative body weight gain after 12weeks; (C) food intake per mouse at every week. (D)Total food intake per mouse after 12 weeks. Data are shown as the mean ± SD (*n* = 6). a, WTH groupversus WTS group; b, WTHC groupversus WTH group; c, KOH group versus KOS group; d, KOHC groupversus KOH group(one-way ANOVA followed by Dunnett’s test, *p* < 0.05).

As shown in Figs. 1A and 1B, the mice fed an HFD gained mass steadily over time and had higher body mass gain (WT mice, by 90 ± 3% and KO mice, by 85 ± 5%) than SLD-fed mice (WT mice, 43 ± 2% and KO mice, 37 ± 2%) after 12 weeks (*p* < 0.05). Intragastrical administration of CAP significantly suppressed the HFD-induced body mass gain(Fig. 1B, *p* < 0.05), with WT mice fed the HFD+CAP (by 39%) showing a greater effect than KO mice fed the same diet (by 21%). Average food intake of HFD+CAP-fed mice was lower than HFD-fed mice in every week (respectively for WT and KO mice, Fig. 1C). About 12 weeks after the feeding intervention commenced, total food intake was lower in the HFD+CAP-fed mice than HFD-fed mice(respectively for WT and KO mice, *p* < 0.05) (Fig. 1D).

### CAP improves glycemic control and dyslipidemia in HFD-fed mice

The effects of intragastrical administration of CAP on biochemical parameters are shown in Table 2. Plasma concentrations of glucose, TGs, cholesterol, and insulin were higher in the HFD-fed WT and KO mice than in the SLD-fed WT and KO mice (*p* < 0.05) (Table 2). The HFD+CAP-fed WT and KO mice had markedly lower fasting glucose (by 41and 28%, respectively) and insulin (by 27and 15%, respectively) than the control HFD-fed mice, restoring the values to those of the SLD group. The HFD+CAP-fed WT and KO mice also exhibited significantly lower plasma TG, plasma total cholesterol, high density lipoprotein cholesterol (HDL-C), and low-density lipoprotein cholesterol (LDL-C)than the control HFD-fed mice (*p* < 0.05) (Table 2).

**Table 2:** Effects of capsaicin on plasma parameters. Data are expressed as mean ± SD (*n* = 6).

Parameters	WTS	WTH	WTHC	KOS	KOH	KOHC
Glucose (mmol/L)	4.76 ± 0.34	9.39 ± 0.13^a^	5.51 ± 0.80^b^	4.66 ± 0.63	8.24 ± 0.21^c^	5.95 ± 0.28^d^
Insulin (pmol/L)	14.02 ± 1.06	20.62 ± 1.33^a^	15.07 ± 1.18^b^	16.03 ± 0.40	21.98 ± 1.61^c^	18.80 ± 1.08^d^
Plasma TG (mmol/L)	0.57 ± 0.07	0.85 ± 0.10^a^	0.61 ± 0.05^b^	0.42 ± 0.12	0.86 ± 0.14^c^	0.64 ± 0.14^d^
Plasma TC (mmol/L)	2.70 ± 0.20	3.79 ± 0.13^a^	2.52 ± 0.17^b^	2.34 ± 0.22	3.41 ± 0.30^c^	2.69 ± 0.30^d^
HDL-Cholesterol (mmol/L)	1.11 ± 0.10	1.66 ± 0.23^a^	0.90 ± 0.13^b^	0.96 ± 0.10	1.45 ± 0.13^c^	1.03 ± 0.18^d^
LDL-Cholesterol (mmol/L)	1.50 ± 0.03	2.06 ± 0.12^a^	1.54 ± 0.13^b^	1.27 ± 0.12	1.90 ± 0.06^c^	1.61 ± 0.04^d^

WTS, WT mice fed with low-fat diet; WTH, WT mice fed with high-fat diet; WTHC, WT mice fed with high-fat diet and CAP; KOS, KO mice fed with low-fat diet; KOH, KO mice fed with high-fat diet; KOHC, KO mice fed with high-fat diet and CAP

a WTH groupversus WTS group

b WTHC groupversus WTH group

c KOH groupversus KOS group

d KOHC groupversus KOH group(one-way ANOVA followed by Dunnett’s test, *p* < 0.05).

**Fig. 2:**
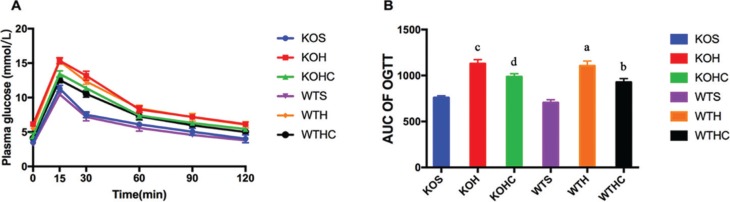
Effects of CAP on glucose tolerance. (A) Glucose tolerance test; (B) AUC of OGTT. Results are represented as mean ± SD (*n* = 6). WTH, WT mice fed with high-fat diet; WTHC, WT mice fed with high-fat diet and CAP; KOS, KO mice fed with low fat diet; KOH, KO mice fed with high-fat diet; KOHC, KO mice fed with high-fat diet and CAP. AUC, area under the curve (0–120 min). a, WTH group versus WTS group; b, WTHC group versus WTH group; c, KOH group versus KOS group; d, KOHC group versus KOH group(one-way ANOVA followed by Dunnett’s test, *p* < 0.05).

A glucose tolerance test was performed during week 11 of the experiment, and glucose tolerance was quantified as the area under the curve (AUC) integrated from 0 to 120 min. The HFD-fed WT and KO mice exhibited slightly impaired glucose tolerance, having higher glucose levels at 15–120 min than SLD-fed WT and KO mice (*p* < 0.05, Fig. 2A). Compared to the HFD-fed WT and KO mice, HFD+CAP-fed WT and KO mice had a smaller (*p* < 0.05) glucose AUC (by 16 and 13%, respectively; Fig. 2B), suggesting better glycemic control than in the HFD-fed mice. These findings confirm that intragastrical administration of CAP helped prevent obesity in HFD-fed mice, and the effect of which is lessened without TRPV1 channel activation.

### CAP induces changes in the composition of the gut microbiota

To assess the impact of intragastrical administration of CAP on the gut microbiota community, various V4 regions of the 16S ribosomal RNA genes extracted from the fecal samples of SLD, HFD, and HFD+CAP-fed groups were sequenced on the Illumina MiSeq platform, and the results are presented as OTUs, using a 97% homology cut-off value (The sequence data is in Appendix A).

**Fig. 3:**
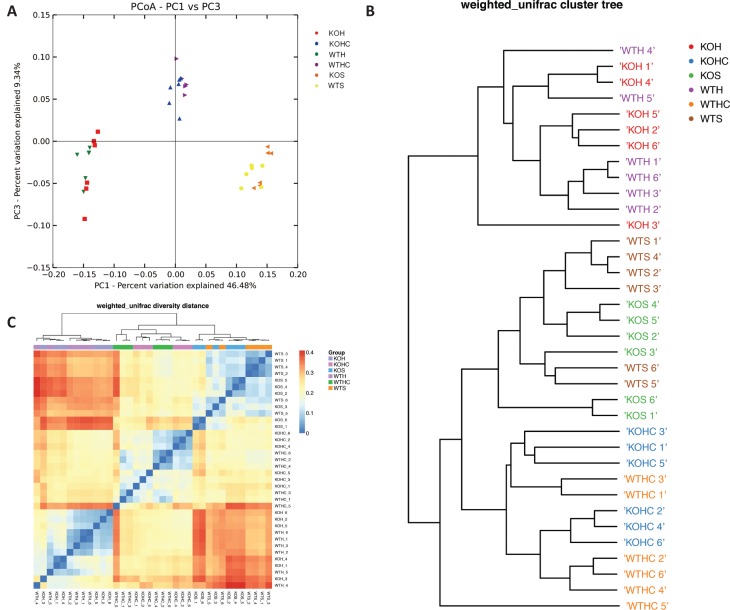
CAP modulated the structure of the gut microbiota. (A) Principal coordinate analysis (PCoA) and (B) sample clustering results of the weighted UniFrac distances of microbial 16S rRNA sequences from the V4 region in fecal samples, (C) heat map representation of hierarchical cluster. WTH, WT mice fed with high-fat diet; WTHC, WT mice fed with high-fat diet and CAP; KOS, KO mice fed with low fat diet; KOH, KO mice fed with high-fat diet; KOHC, KO mice fed with high-fat diet and CAP.

Differences in bacterial community structures are apparent in PCoAs (Fig. 3A). PCoAs revealed a distinct clustering of microbial species among the SLD, HFD, and HFD+CAP groups, along the primary ordination axis. KO mice fed with SLD, HFD, or HFD+CAP had microbial populations similar to WT mice fed with SLD, HFD, or HFD+CAP, respectively. The HFD-fed WT and KO groups clustered together at one end of the axis, while the SLD-fed WT and KO groups clustered at the opposite end, and the HFD+CAPWT and KO groups formed a distinct cluster in the middle. Hierarchical clustering showed that the microbial communities in the feces of the HFD+CAP WT and KO groups were more closely related to those of the SLD WT and KO groups than those of the HFD groups (Fig. 3B).

The relative abundances of the taxa, which gave a *p*-value of < 0.05 by differential expression analysis (nonparametric ANOVA with FDR correction), were then expressed as a heat map (Fig. 3C), including hierarchical clustering (HCN). Hierarchical clustering clearly separated the HFD WT and KO groups as a single cluster from the other two groups, which formed two clusters within a clade.

**Fig. 4:**
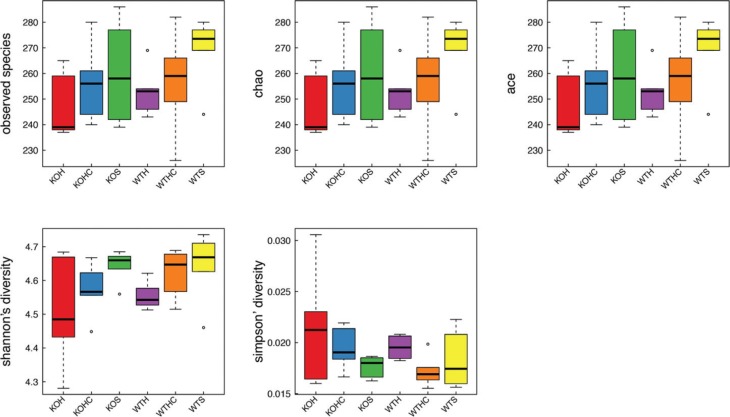
CAP modulated the diversity of the gut microbiota. WTH, WT mice fed with high-fat diet; WTHC, WT mice fed with high-fat diet and CAP; KOS, KO mice fed with low fat diet; KOH, KO mice fed with high-fat diet; KOHC, KO mice fed with high-fat diet and CAP (alpha diversity analysis at the OUT level calculated on denoised sequences of mouse fecal microbiota.)

To assess intestinal microbial community structure, richness and evenness were calculated (Fig. 4). At the OTU level, the intestines of HFD-fed WT and KO mice were populated with fewer species than those of SLD-fed WT and KO mice. The intestines of HFD+CAP-fed WT and KO mice contained a larger number of species than those of HFD-fed WT and KO mice, but a smaller number than those of the SLD WT and KO mice. Diversity, assessed using Shannon’s richness index, was significantly lower in the HFDWT group than in the SLDWT group. The HFD+CAPWT group had a higher value for Shannon’s index than the HFD WT group, but this did not reach the level of the SLD WT group. When assessed using Simpson’s evenness index, diversity was also greater in the HFD WT group than the SLD WT group. Simpson’s index for the HFD+CAP WT group was lower than for the HFD WT group, but not to the same extent as for the SLD WT group. The KO mice demonstrated trends similar to the WT mice in their diversity, assessed using both Shannon’s richness and Simpson’s evenness indices, but the differences between diet groups were less than those for the WT mice.

**Fig.5:**
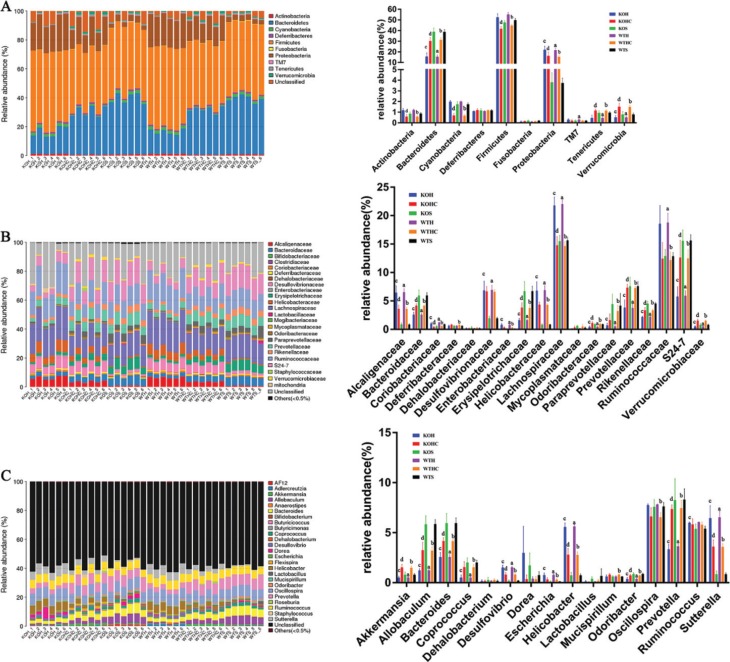
CAP modulated the composition of the gut microbiota. (A) Phylum-level, (B)family-level, and (C) genus-level taxonomic distributions of the microbial communities in fecal. Data are expressed as mean ± SD (*n* = 6). WTH, WT mice fed with high-fat diet; WTHC, WT mice fed with high-fat diet and CAP; KOS, KO mice fed with low fat diet; KOH, KO mice fed with high-fat diet; KOHC, KO mice fed with high-fat diet and CAP. a, WTH groupversus WTS group; b, WTHC groupversus WTH group; c, KOH group versus KOS group; d, KOHC groupversus KOH group(one-way ANOVA followed by Dunnett’s test, *p* < 0.05).

To assess specific changes in the gut microbiota, we compared the relative abundances of the predominant taxa identified from sequencing of the fecal microbial populations of mice in each of the three diet groups (Fig. 5). Significant differences in the composition of the gut microbiota were found at all taxonomic levels. We only focused on the same trends for the KO mice and the WT mice, as shown below.

At the phylum level, the microbiota from the HFD-fed mice contained relatively few Bacteroidetes, Tenericutes, and Verrucomicrobia compared with that from the SLD-fed mice (Fig. 5A). However, the relative abundance of Proteobacteria was markedly higher in the HFD-fed mice and there was a trend towards a larger population of Firmicutes, Actinobacteria, and Cyanobacteria. In marked contrast to the HFD group, the HFD+CAP diet-associated microbial community contained a significantly larger relative abundance of Bacteroidetes, Tenericutes and Verrucomicrobia, and a much lower relative abundance of Proteobacteria, Actinobacteria, Cyanobacteria, and Firmicutes.

At the family level, compared with the SLD, the fecal microbiota of the HFD-fed mice contained fewer Verrucomicrobiaceae, Odoribacteraceae, Paraprevotellaceae, Rikenellaceae, Prevotellaceae, Bacteroidaceae, S24-7, and Erysipelotrichaceae, and more Alcaligenaceae, Coriobacteriaceae, Desulfovibrionaceae, Helicobacteraceae, Ruminococcaceae, and Lachnospiraceae (Fig. 5B). Mice fed the HFD+CAP differed greatly from the HFD-fed mice with respect to the significantly higher relative abundances of Bacteroidaceae, Erysipelotrichaceae, Odoribacteraceae, Prevotellaceae, Rikenellaceae, Verrucomicrobiaceae, S24-7, and much lower relative abundance of Alcaligenaceae, Coriobacteriaceae, Helicobacteraceae, Lachnospiraceae, Ruminococcaceae.

At the genus level, HFD-feeding was associated with a larger relative abundance of *Helicobacter*, *Desulfovibrio*, *Escherichia*, and *Sutterella*, but smaller populations of *Akkermansia*, *Prevotella*, *Bacteroides*, *Odoribacter*, *Allobaculum*, and *Coprococcus* (Fig. 5C). Feeding the HFD+CAP was associated with the presence of 51% fewer *Helicobacter* in WT mice and 50% fewer in KO mice,45 and 44% fewer *Sutterella*, 48 and 47% fewer *Desulfovibrio*, and 61 and 60% (but no significant difference) fewer *Escherichia*, respectively. It was also associated with threefold populations of Akkermansia in both WT and KO mice , 2.1-and 2.2-fold populations of Prevotella, 1.6- and 1.6-fold populations of *Bacteroides*, 2.6- and 2.7-fold populations of *Allobaculum*, and3- and 3.1-fold populations of *Coprococcus*, and 1.9- and 2-fold (but no significant difference) populations of *Odoribacter,* respectively.

### CAP affects fecal SCFA excretion

The effects of intragastric administration of CAP on fecal SCFA excretion are shown in Table 3. Fecal acetate and propionate concentrations were lower in the HFD-fed WT and KO mice (*p* < 0.05), than in the SLD-fed WT and KO mice. Intragastrical administration of CAP attenuated these differences (*p* < 0.05). There were no significant differences in the concentrations of butyrate among the groups.

**Table 3:** Effects of CAP on fecal SCFA excretion. Data are expressed as mean ± SD (*n* = 6).

SCFA (mg/g)	WTS	WTH	WTHC	KOS	KOH	KOHC
Acetate	15.86 ± 1.95	10.71 ± 1.24^a^	13.82 ± 1.72^b^	15.95 ± 2.03	11.27 ± 2.65^c^	14.23 ± 1.96^d^
Propionate	1.90 ± 0.14	1.58 ± 0.19^a^	1.79 ± 0.13^b^	1.91 ± 0.24	1.55 ± 0.16^c^	1.75 ± 0.06^d^
Butyrate	1.08 ± 0.21	1.02 ± 0.18	1.01 ± 0.19	1.21 ± 0.14	1.10 ± 0.14	1.07 ± 0.15

WTS, WT mice fed with low-fat diet; WTH, WT mice fed with high-fat diet; WTHC, WT mice fed with high-fat diet and CAP; KOS, KO mice fed with low-fat diet; KOH, KO mice fed with high-fat diet; KOHC, KO mice fed with high-fat diet and CAP

a WTH groupversus WTS group

b WTHC groupversus WTH group

c KOH groupversus KOS group

d KOHC groupversus KOH group(one-way ANOVA followed by Dunnett’s test, *p* < 0.05).

### mRNA expression of FFAR2, gut hormone peptides, gut hormone peptides receptor, and neuropeptides

**Fig. 6:**
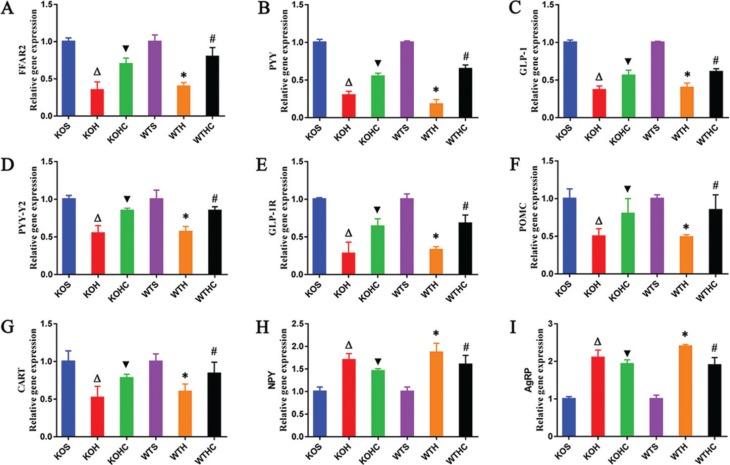
CAP regulated mRNA expressions of FFAR2, gut hormone peptides, gut hormone peptides receptor, and neuropeptides. (A) FFAR2 gene expressions,(B) PYY gene expressions, (C) GLP-1 gene expressions in the colon; (D) PYY-Y2 gene expressions, (E) GLP-1R gene expressions, (F) POMC gene expressions,(G) CART gene expressions, (H) NPY gene expressions, and (I) AgRP gene expressions in the hypothalamus. Results are represented as mean ± SD (*n* = 6). WTH, WT mice fed with high-fat diet; WTHC, WT mice fed with high-fat diet and CAP; KOS, KO mice fed with low-fat diet; KOH, KO mice fed with high-fat diet; KOHC, KO mice fed with high-fat diet and CAP. a, WTH groupversus WTS group; b, WTHC groupversus WTH group; c, KOH group versus KOS group; d, KOHC groupversus KOH group( one-way ANOVA followed by Dunnett’s test, *p* < 0.05).

The mRNA expression of FFAR2, PYY, and GLP-1 in the colon was significantly lower in the HFD-fed WT and KO mice than in the SLD fed WT and KO mice (*p* < 0.05). Intragastrical administration of CAP supplementation (WT and KO) significantly increased the mRNA expression of FFAR2, PYY, and GLP-1 (*p* < 0.05) (Figs. 6A–6C) compared to the HFD-fed WT and KO mice. In the HFD+CAP fed WT and KO mice, the expression of PYY-Y2, GLP-1R, POMC, and CART (Figs. 6D–6G) was significantly higher (*p* < 0.05), whereas the expression of NPY and AgRP (Figs. 6H–6I) in the hypothalamus was lower than that of the HFD-fedWT and KO mice (*p* < 0.05).

## Discussion

CAP has been shown to protect against body mass gain in rodents and other species (5, 6). Likewise, in our study, we observed significantly lower gains in body mass, TGs, cholesterol, and insulin, and a smaller glucose AUC in HFD+CAP-fed WT and KO mice compared with HFD-fed WT and KO mice. Zhang et al. showed that CAP did not prevent obesity in HFD-fed male TRPV1 KO mice, but CAP-induced calcium influx through activation of TRPV1 channels prevents adipogenesis and obesity in male WT mice (5). In our study, we observed that CAP suppressed body mass gain in TRPV1 female KO mice, though this effect was greater in female WT mice, which is not consistent with the findings of Zhang et al. The first reason for this could be that male C57/BL6 mice are more vulnerable to HFD-induced obesity and metabolic syndrome compared with female C57/BL6 mice fed HFD (19, 20). So, obesity induced by HFD in male C57/BL6 mice is not easily prevented by CAP. Moreover, limiting weight gain can be achieved not only by reducing adipogenesis, but also by reducing intestinal lipid absorption and/or lowering food intake. Rohm et al. showed that CAP reduced energy intake, had a hypolipidemic effect, and decreased free fatty acid uptake without TRPV1 activation in Caco-2 cells (21). Here, we show that intragastrical administration of CAP significantly inhibits HFD-induced body mass gain, but this effect is smaller when TRPV1 channels are not activated. Therefore, the anti-obesity effect of CAP may involve an alternative mechanism needing further studies, such as the effect of altered gut microbiota.

The interaction between dietary components and intestinal microorganisms shapes the composition of the gut microbiota, and has a significant impact on host metabolism. Many studies have shown that aberrant gut microbiota composition is associated with the development of obesity the metabolic syndrome in humans (8). Shen et al. showed that the proportions of *Bacteroides*, *Coprococcus*, *Prevotella*, and *Akkermansia* were significantly higher in HFD-fed mice after feeding of CAP (13). In agreement with these findings, we observed that the relative abundances of *Bacteroides*, *Coprococcus*, *Prevotella*, and *Akkermansia* in HFD+CAP-fed WT mice were 1.6-, 3-, 2.1-, and 3-fold changed, respectively, compared to those of HFD-fed WT mice. The relative abundances of these genera in HFD+CAP-fed KO mice were also higher: by 1.6-, 3.1-, 2.2-, and 3-fold, respectively. Many studies have also shown higher ratios of Firmicutes to Bacteroidetes in the gut of obese humans and animals (22), whereas a higher relative abundance of Bacteroidetes may be beneficial for weight loss in obese humans (8). Baboota et al. found that the relative abundance of *Akkermansia* was higher in HFD+CAP-fed mice, using qPCR to detect differences in the abundance of seven gut microbes (12). Many other microbiome studies, such as those evaluating the effects of black tea, polyphenols, bamboo shoot fiber, or cranberry, have also shown that a larger population of *Akkermansia* might mediate some of the anti-obesity effects (7, 23–25). Greater levels of fiber consumption lead to improvements in glucose profiles, in association with an increase in *Prevotella* abundance. Kovatcheva-Datchary et al. found that an increase in the population of *P. copri* alone, without a change in diet, could improve glucose tolerance in mice and humans (26).

Unique to our study was the finding that the markedly lower relative abundances of *Odoribacter*, *Allobaculum,* and S24-7 induced by HFD-feeding, were suppressed by CAP. *Odoribacter*, a genus of mucin-degrading bacterial, has been found to be associated with a healthy lipid metabolism (27). Etxeberria et al. found that the anti-obesity effects of Pterostilbene induced in Zucker rats could be associated with an enrichment of *Akkermansia* and *Odoribacter* (28). A large population of *Allobaculum* spp. and their relatives, which consume simple sugars rapidly, leads to rapid energy harvesting and favors adiposity (29). Van Hul et al. observed a smaller population of *Desulfovibrio* and *Lactococcus*, and larger populations of *Allobaculum* and *Roseburia*, when they assessed the metabolic effects of polyphenol-containing extracts from grape pomace in C57BL/6J mice fed an HFD for 8 weeks (30). The relative abundances of both S24-7 and *Allobaculum* were reduced by the introduction of obesogenic diets, but were increased if a greater proportion of fermentable fiber was added to the diet, which can lead to improvements in glucose tolerance (31, 32). Thus, changes in the abundance of *Odoribacter*, *Allobaculum* and S24-7 may be involved in the effects of treatments for glucose intolerance and obesity. A study by Shen et al. demonstrated the proportions of *Bacteroides*, *Coprococcus*, *Prevotella*, and *Akkermansia* to be significantly higher in HFD-fed mice after CAP feeding. The similar results obtained in the present study further confirmed this finding. However, we found that CAP feeding also affected other bacterial populations, such as *Odoribacter*, Allobaculum, and S24-7. Kang et al. showed that S24-7 is the key bacterial family that largely contributes to the HFD group for inducing chronic low-grade inflammation (14). However, many other studies have shown that chronic low-grade inflammation is mainly induced by Proteobacteria, rather than S24-7, in the intestinal tract (33–39), whereas S24-7 has been reported to be effective in the treatments of glucose intolerance and obesity (31, 32). Shen et al. believed the anti-obesity effect of CAP to be associated with an increase in the abundance of the gut bacterium *Akkermansia muciniphila* (13). Although *Akkermansia* has been widely reported to have anti-obesity effects (7, 12, 23–25), our study does not support this finding of Shen et al. (13). Our data suggested that CAP affects the abundance of many different bacteria, which all affect whole body metabolism. Therefore, we believe that CAP affects the abundance of a variety of bacterial populations to induce its anti-obesity effects.

Unique to our study, the greater relative abundances of *Sutterella*, *Desulfovibrio*, *Escherichia*, and *Helicobacter* induced by HFD-feeding, were suppressed by CAP. An increase in the abundance of Proteobacteria in the intestinal microbiota can activate immune signaling pathways, leading to a chronic, low-grade inflammatory response (36). The relative abundance of Proteobacteria in a healthy gastrointestinal tract varies from 2 to 5%, but can be up to 15% in the presence of metabolic disorders or intestinal inflammation (38). Chronic, low-grade inflammatory responses are associated with higher risks of obesity and obesity-related diseases (34), meaning that the abundance of Proteobacteria in the intestinal tract may have a significant impact on host metabolism. *Desulfovibrio* is a sulfate-reducing bacteria that stimulates gut immune responses and contributes to inflammation (35). Some data suggest that development of colitis involves an increase in *Desulfovibrio* prevalence, likely in association with the generation of hydrogen sulfide (37). Increased populations of gut *Escherichia* may also contribute to intestinal inflammation as a result of generation of lipopolysaccharides (33). *Sutterella*, a genus of Proteobacteria, has been suggested to play a part in the pathogenesis of inflammatory bowel disease, which is associated with a greater risk of obesity. Many studies have shown that *Helicobacter* infection is significantly and positively associated with the risk of being overweight and obesity (39), and the mechanism involved may be the same as that for *Sutterella*. Thus, CAP may exert its anti-obesity effect by reducing the relative abundances of Proteobacteria spp., such as *Desulfovibrio, Escherichia, Sutterella*, and *Helicobacter*, which can lead to a chronic, low-grade inflammatory response.

We believe that the alterations in gut microbiota caused by CAP are important for its anti-obesity effect. However, the change led by the alterations in gut microbiota is more important, such as change in the concentration of SCFAs in the intestines. SCFAs are end-products of the microbial fermentation of dietary fibers and resistant starch, and may play a role in the prevention and treatment of obesity (9–11, 40, 41). It has been demonstrated that fecal concentrations of acetate in diet-induced obese mice fed a HFD containing 45% calories from fat were lower than those in control mice fed a diet containing 10% calories from fat (42), which is consistent with the findings of the present study. In our study, fecal acetate and propionate concentrations in HFD+CAP-fed WT and KO mice were lower than in SLD-fed WT and KO mice (*p* < 0.05), but higher than in HFD-fed WT and KO mice (*p* < 0.05). Lu et al. showed that acetate, propionate, and butyrate all suppress HFD-induced weight gain, with acetate having the greatest effect (11). Lin et al. found that supplementation with butyrate and propionate protected against diet-induced obesity by lowering food intake and increasing gut hormone secretion (43). Thus, CAP may prevent obesity by regulating the concentration of SCFAs in the intestines.

After considering the differences in the gut microbiota alongside those in SCFA concentrations, we noticed that the bacterial species that were more abundant in the gut of CAP-treated mice, such as *Akkermansia*, *Prevotella*, *Allobaculum*, S24-7, *Bacteroides*, and *Coprococcus*, can all perform fermentation reactions that produce acetate or propionate, which likely explains the differences in SCFA content between groups. Murugesan et al. hypothesized that the gut microbiota plays an important role in weight regulation, mediated by the effects of SCFAs on energy balance (40). Therefore, regulation of the gut microbiota and thus the concentration of intestinal SCFAs may be involved in the anti-obesity effect of CAP.

The G-protein-coupled receptors GPR43 and GPR41 have been identified as SCFA receptors, and have since been renamed FFAR2 and FFAR3, respectively (44, 45). FFAR2 is activated preferentially by acetate and propionate, while FFAR3 is activated preferentially by propionate and butyrate. FFAR2 and FFAR3 co-localize with colonic L-cells and stimulate the release of peptide tyrosine (PYY), and glucagon-like peptide 1(GLP-1) (46, 47). However, Tolhurst et al. and Psichas et al. report that FFAR2, but not FFAR3, plays a primary role in stimulating gut hormone release *in vivo* (47, 48).

Colonic administration of propionate stimulates the secretion of both GLP-1 and PYY in rodents, but this effect was significantly attenuated in FFAR2 KO animals. In the present study, fecal acetate and propionate concentrations were different between groups, which would be expected to have effects predominantly via FFAR2, which may explain why we only detected the expression of FFAR2. We found that dietary CAP supplementation in HFD-fed WT and KO mice resulted in increased expression of FFAR2 in the colon, which was positively associated with the expression of both PYY and GLP-1. GLP-1 has been found to cross the blood–brain barrier and specifically act on the hypothalamus to induce weight loss and reduce food intake (49, 50). PYY induces satiety and reduces food intake (51), and our data show that PYY-Y2 and GLP-1R expressions were also significantly higherin the hypothalamus of CAP-supplemented HFD-fed mice (both WT and KO), differences that also showed associations with the expression of PYY and GLP-1. van Avesaat et al. reported that intraduodenal infusion of CAP significantly increases satiety but does not affect plasma concentrations of GLP-1 or PYY (52), which is contradictory to our results of expression of PYY and GLP-1. The reason for this may be that the experiment of van Avesaat et al. was not conducted for long enough to affect the composition of the gut microbiota that regulates the concentration of SCFAs, which stimulates the secretion of both GLP-1 and PYY (52).

The arcuate nucleus (ARC) of the hypothalamus is thought to be pivotal in appetite regulation. Within the ARC, one group of neurons expresses proopiomelanocortin (POMC) and amphetamine-regulated transcript (CART), whose activation reduces appetite (53), and another group of neurons expresses neuropeptide Y (NPY) and agouti-related peptide (AgRP), whose activation increases appetite (54). PYY and GLP1 affect appetite and satiety by suppressing NPY and AgRP expression and activating POMC and CART expression in the ARC. In the present study, compared with HFD-fed WT and KO mice, the HFD+CAP-fed WT and KO mice showed significantly higher POMC and CART expression and significantly lower NPY and AgRP expression in the hypothalamus (*p* < 0.05). Thus, the addition of dietary CAP may increase satiety and reduce appetite by enhancing PYY and GLP-1 expression, resulting in an inhibition of neurons expressing NPY and AgRP, and an activation of neurons expressing POMC and CART in the ARC. We found this possible mechanism of CAP’s anti-obesity effect by measuring the expression of FFAR2, PYY, GLP1, PYY-Y2, GLP-1R, POMC, CART, NPY, and AgRP. However, this possible mechanism still needs to be verified by further research, such as quantifying the plasma PPY-1, GLP-1 levels, and the western blot result of POMC, CART, NPY, and AgRP protein, which is also the weaknesses of the study.

## Conclusions

**Fig. 7:**
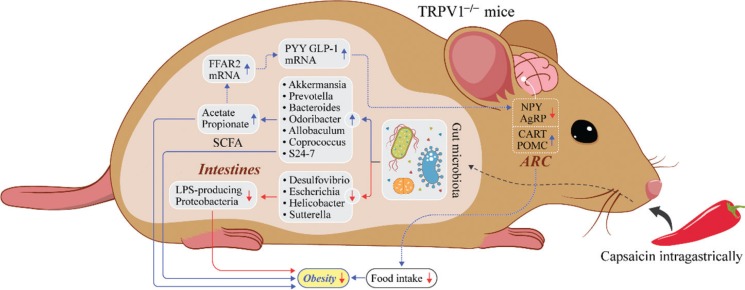
Anti-obesity effect mechanism of CAP in this study.

Intragastrical administration of CAP is effective in suppressing the excessive body weight gain induced by HFD-feeding in female mice and reducing food intake regardless of TRPV1 channel activation. The effect of CAP on weight gain and food intake may be mediated by an increase in the relative abundances of *Bacteroides*, *Coprococcus*, *Prevotella*, *Akkermansia*, *Odoribacter, Allobaculum,* and S24-7, which are beneficial for weight loss in obese mice. This change in the relative abundances of those SCFA-producing bacterial species results in enhanced acetate and propionate production in the gut, which also are beneficial for energy balance in obese mice. The effect of CAP may be mediated by reducing the relative abundances of Proteobacteria spp., such as *Desulfovibrio, Escherichia, Sutterella*, and *Helicobacter*, which can generate a chronic, low-grade inflammatory response associated with higher risks of obesity. We also found a possible mechanism of CAP’s anti-obesity effect by enhancing PYY and GLP-1excretion in intestinal epithelial cells, which suppress NPY- and AgRP-expressing neurons and activate POMC- and CART-expressing neurons in the ARC of the hypothalamus, resulting in lower food intake. However, this possible mechanism still needs to be verified by further research (Fig. 7).

## Appendix A. Supplementary data

The sequence data supporting the results of this article are available in the NCBI Sequence Read Archive under SRA accession number SRR6384129-SRR6384164.
